# The Role of Apoptosis and Ferroptosis in Primary Mitochondrial Diseases: Mechanisms and Pathogenesis

**DOI:** 10.3390/ijms27135931

**Published:** 2026-07-01

**Authors:** Anastasia Kolotova, Alexandr Shestopalov, Sergey Kutsev

**Affiliations:** 1 Research Centre for Medical Genetics, 1 Moskvorechye St., 115478 Moscow, Russia; 2 Pirogov Russian National Research Medical University (Pirogov RNRMU), 70, Nizhnyaya Pervomayskaya Str., 105203 Moscow, Russia

**Keywords:** mitochondrial diseases, apoptosis, ferroptosis, regulated cell death, oxidative stress, lipid peroxidation, iron metabolism, tissue specificity, molecular crosstalk, mitochondria

## Abstract

Mitochondrial diseases have traditionally been viewed as energy deficiencies, but current evidence positions mitochondria as central regulators of multiple cell death pathways. This review systematically analyzes the molecular mechanisms of apoptosis and ferroptosis in the context of both primary mitochondrial diseases—caused by mutations in mtDNA or nuclear DNA directly affecting oxidative phosphorylation—and secondary mitochondrial dysfunction associated with broader pathological conditions. Apoptosis is an energy-dependent process characterized by mitochondrial outer membrane permeabilization, cytochrome c release, and caspase cascade activation, whereas ferroptosis involves iron-dependent lipid peroxidation, glutathione depletion, and inactivation of glutathione peroxidase 4 (GPX4), leading to accumulation of oxidized phospholipids predominantly in endoplasmic reticulum and plasma membranes; mitochondrial ultrastructural changes—including volume reduction and cristae loss—represent characteristic morphological features of ferroptosis rather than its primary site of initiation. Key findings reveal that reactive oxygen species overproduction, disruption of reducing equivalent metabolism, iron dyshomeostasis, and calcium overload simultaneously prime cells for both death pathways. Cytochrome c, p53, and BCL-2 family proteins serve as integration hubs, with cardiolipin peroxidation and phospholipid composition influencing pathway switching. Tissue specificity is pronounced in primary mitochondrial diseases: retinal ganglion cells in Leber’s hereditary optic neuropathy, cardiomyocytes in mtDNA-associated cardiomyopathies, and hepatocytes in mtDNA depletion syndromes exhibit distinct dominant death pathways. It should be noted, however, that for many conditions discussed, the evidence for ferroptosis involvement relies on indirect markers—such as lipid peroxidation products, decreased GPX4, and iron deposition—rather than on pharmacological rescue with ferrostatin-1 or liproxstatin-1 and rigorous exclusion of alternative death modalities; this limitation is discussed critically throughout the review. Diagnostic criteria combining morphological, biochemical, and pharmacological tools enable differentiation of death pathways. The review concludes that combined inhibition—using mitochondria-targeted antioxidants, GPX4 modulators, iron chelators, and mPTP blockers—together with personalized diagnostic algorithms offers the most promising therapeutic strategy. Understanding the apoptosis–ferroptosis crosstalk is essential for developing targeted interventions in mitochondrial diseases.

## 1. Introduction

### 1.1. Evolution of Concepts of the Pathogenesis of Mitochondrial Diseases

Mitochondrial diseases in the classical sense have long been viewed as a simple “energy deficit,” where the central idea was impaired adenosine triphosphate (ATP) production due to defects in oxidative phosphorylation. However, the current understanding of these diseases has shifted toward recognizing them as complex pathologies in which mitochondria act as central regulators of multiple cell death signaling pathways, such as apoptosis, necroptosis, ferroptosis, pyroptosis, and autophagy [1,2]. Mitochondrial diseases are driven by complex molecular crosstalk with other cellular organelles, in which mitochondrial damage links metabolic stress to cell death pathways, leading not only to energy deficiency but also to aggravation of tissue injury [1,3]. Thus, mitochondrial dysfunction results in both energy deficit and increased production of reactive oxygen species (ROS), disruption of calcium homeostasis, and opening of the mitochondrial permeability transition pore (mPTP), thereby initiating various mechanisms of programmed cell death [2,4].

Mitochondrial diseases possess specific features. One of these is tissue specificity of manifestations, which arises from the differing sensitivity of cell types in tissues to particular death pathways activated by mitochondrial dysfunction, resulting in varying disease phenotypes in organs such as the heart, brain, and lungs [1,4]. In addition to tissue specificity, these diseases exhibit considerable genetic heterogeneity due to mutations in both mitochondrial DNA (mtDNA) and nuclear DNA (nDNA) affecting key mitochondrial processes such as oxidative phosphorylation (OXPHOS) and metabolic pathways [5,6]. A distinctive feature of the genetics of mitochondrial diseases caused by mtDNA mutations is heteroplasmy—the simultaneous presence of mutant mtDNA and wild-type mtDNA in cells—so that the severity of the disease varies with the proportion of mutant mtDNA. Heteroplasmy is characterized by a threshold effect that depends on tissue type and mutation: for clinical symptoms to appear, the mutant load must exceed a critical level at which cellular energy homeostasis is disrupted [7,8,9]. Mitochondrial diseases are often maternally inherited, and heteroplasmy levels influence tissue-specific vulnerability and phenotypic heterogeneity [10,11].

In this review, the term “mitochondrial diseases” is used in two contexts that must be distinguished. Primary mitochondrial diseases (PMDs) are genetically determined disorders caused by mutations in mtDNA or nDNA that directly affect OXPHOS function (LHON, Leigh syndrome, MELAS, mtDNA depletion syndromes). Secondary mitochondrial dysfunction refers to impairment of mitochondrial function as a component of multifactorial pathology, where mitochondria are not the primary target (Parkinson’s disease with *PINK1/Parkin* mutations, CNS injury, myocardial ischemia–reperfusion, doxorubicin cardiotoxicity). Data on the roles of ferroptosis and apoptosis in these two groups have been obtained from fundamentally different experimental systems and carry different levels of evidence.

The accumulation of data on the signaling role of mitochondria and the fact that many mitochondrial disorders are realized through the induction of apoptosis have led to the consideration of these processes within the framework of regulated cell death (RCD).

### 1.2. The Concept of Regulated Cell Death in Modern Biology

In modern biology, regulated cell death refers to genetically determined molecular processes that require energy and are controlled by the organism, distinguishing them from accidental or uncontrolled cell death.

According to the classification of the Nomenclature Committee on Cell Death (NCCD), based on molecular mechanisms, RCD encompasses a variety of pathways—intrinsic and extrinsic apoptosis, mitochondrial permeability transition-driven necrosis, necroptosis, pyroptosis, ferroptosis, parthanatos, entotic cell death, NETotic cell death, lysosome-dependent cell death, autophagic cell death, immunogenic cell death, cellular senescence, and mitotic catastrophe [12]. Each type of death is characterized by specific signaling cascades and key effector molecules, such as caspases in apoptosis or *RIPK3/MLKL* in necroptosis [13,14,15]. All these pathways are essential for organismal development, maintenance of tissue homeostasis, immune system function, and removal of damaged or infected cells; dysregulation of these pathways leads to the development of malignant tumors, inflammatory conditions, and neurodegeneration [13,15,16].

According to current concepts, there is considerable interconnection and overlap between forms of RCD, underscoring the need for and importance of molecular markers for each type of cell death [17,18]. For instance, lipid peroxidation is regarded as a common biochemical phenomenon for ferroptosis and pyroptosis, highlighting the existence of shared mechanisms. It can be stated that the concept of RCD as a whole has evolved into a complex network of interconnected pathways regulating cell fate, which has implications for therapeutic targeting in various pathologies [14,16,19].

### 1.3. Aim and Objectives of the Review

Apoptosis and ferroptosis represent two fundamentally different forms of RCD, differing in their molecular prerequisites, biochemical cascades, and morphological features [20]. Apoptosis is an energy-dependent process of cell destruction characterized by increased permeability of the outer mitochondrial membrane, release of cytochrome c, and activation of the caspase cascade, whereas ferroptosis is associated with iron-dependent lipid peroxidation, glutathione depletion, and GPX4 inactivation, leading to the accumulation of oxidized phospholipids primarily in the membranes of the endoplasmic reticulum and the plasma membrane; mitochondrial changes (reduced volume, loss of cristae) are characteristic morphological features, but the primary compartment of damage is the ER [3,21,22]. Cells can undergo both pathways due to common causes, such as ROS accumulation and various types of mitochondrial damage. Thus, fragmentation, loss of membrane potential, and altered metabolism contribute to the initiation of both apoptosis and ferroptosis [2,23]. Furthermore, in the crosstalk characteristic of mitochondria, cytochrome c can act both as a trigger of apoptotic signaling cascades and as a mediator of lipid peroxidation in ferroptosis, emphasizing the role of its binding to cardiolipin as a critically important intersection point [2,3]. The dominant cell death pathway can be determined by tissue specificity: cardiomyocytes in doxorubicin-induced cardiomyopathy often show signs of both apoptosis and ferroptosis, whereas in neuronal cells BID-mediated mitochondrial damage is observed, linking ferroptosis to apoptosis [2,22]. Understanding these interactions is essential for developing targeted therapies that allow modulation of specific cell death pathways depending on the tissue context and disease stage in mitochondrial diseases [1,24]. This literature review aims to systematize current data on the roles of apoptosis and ferroptosis in the pathogenesis of mitochondrial diseases.

## 2. Biochemical and Molecular Bases of Apoptosis, Ferroptosis and Other Forms of RCD

### 2.1. Apoptotic Pathways

#### 2.1.1. Intrinsic (Mitochondrial) Pathway of Apoptosis

Apoptosis is executed through two main pathways: the intrinsic, or mitochondrial, pathway and the extrinsic, or receptor-mediated, pathway, which ultimately converge on the activation of effector caspases.

The intrinsic pathway of apoptosis is mediated by intracellular stress of various origins—DNA damage, oncogene activation, ROS accumulation, excess Ca^2+^, etc.—and involves the following main stages: initiation, including activation of pro-apoptotic proteins, mitochondrial outer membrane permeabilization (MOMP), release of apoptogenic factors, apoptosome formation, and caspase activation [15,25].

The BCL-2 protein family plays a central role in the regulation of apoptosis by controlling MOMP and is divided into three functional groups [26]:

1. Pro-apoptotic effectors (BAX, BAK): these proteins are directly responsible for pore formation in the membrane [26].
2. Anti-apoptotic regulators (BCL-2, BCL-XL, MCL-1, BCL-W, BFL-1): these proteins contain four BH domains (BH1–BH4) and form a hydrophobic groove for interaction with pro-apoptotic proteins [27].
3. BH3-only proteins (sensors): these include BIM, PUMA, BID, BAD, NOXA, BMF, BIK, and HRK [28,29]. They are activated in response to specific stresses and transmit the death signal via two mechanisms:
  - Activators (BIM, PUMA, tBID) can directly bind to BAX and BAK, triggering their activation [12];
  - Sensitizers (BAD, NOXA, HRK) bind to anti-apoptotic proteins, displacing effectors or activators and thereby promoting MOMP [28].

Activated BAX and BAK undergo conformational changes, insert into the membrane, and oligomerize, forming large ring-shaped lipid structures that include BCL-2 protein oligomers, which leads to a radical rearrangement of mitochondrial ultrastructure [26], thus mediating MOMP [30,31]. As a result, MOMP not only triggers caspase activation but can also engage non-lethal signaling pathways, indicating the multifaceted role of mitochondria in determining cell fate [9,11]. MOMP regulation is influenced by protein kinase activity [10] and directly by mitochondrial membrane lipids and resident proteins, confirming the complex interplay between mitochondrial structure and BCL-2 family proteins in the control of apoptosis [32,33].

Through the formed pores, pro-apoptotic proteins are released from the intermembrane space into the cytosol:

- Cytochrome c, which binds to APAF1 in the presence of dATP [34], inducing its oligomerization into a heptameric wheel-shaped complex—the apoptosome [35];
- SMAC/DIABLO and Omi/HtrA2, which neutralize inhibitors of apoptosis (XIAP), freeing caspases to execute the death program unimpeded [25].

Procaspase-9 is activated within the apoptosome via homodimerization and converted into caspase-9, which subsequently triggers the caspase cascade by cleaving and activating executioner caspases, in particular caspase-3 and caspase-7 [12,36], leading to:

- DNA fragmentation through inactivation of ICAD and activation of CAD endonuclease [37];
- Destruction of the nuclear lamina and cytoskeleton, followed by cell shrinkage and membrane blebbing [25];
- Externalization of phosphatidylserine, which ensures the “immunologically silent” removal of the cell by phagocytes—efferocytosis [38].

The final stage of the intrinsic apoptosis pathway is the formation of apoptotic bodies—membrane-bound vesicles containing cellular contents that are released during the terminal disintegration of the cell [39].

Alongside the canonical MOMP-dependent pathway, a caspase-independent mechanism of intrinsic apoptosis exists. Under pronounced ATP depletion or sustained mPTP opening, AIF and endonuclease G are released from mitochondria and translocate directly to the nucleus without engaging the apoptosome complex. AIF induces large-scale DNA fragmentation (~50 kbp), whereas endonuclease G carries out nucleosomal chromatin degradation. This pathway is fundamentally important for understanding cell death in tissues with low caspase levels—for example, in cardiomyocytes and mature muscle fibers.

#### 2.1.2. Extrinsic (Receptor-Mediated) Pathway of Apoptosis

The extrinsic, receptor-mediated pathway of apoptosis is initiated by extracellular stimuli that are recognized by specialized death receptors (DRs) located on the plasma membrane [25]. This mechanism plays a critical role in maintaining immune homeostasis and eliminating infected or tumor cells [38].

The key components of pathway activation are death receptors (DRs) belonging to the tumor necrosis factor receptor (TNFR) superfamily [40], which share a cysteine-rich extracellular domain and an intracellular death domain (DD), as well as TRAIL-R1/R2 receptors [41]. Their activation occurs upon binding to specific ligands (FasL, TNFα, TRAIL), leading to receptor oligomerization and a conformational change in their intracellular death domains (DD).

Following activation, the receptor DD domains recruit adaptor proteins. In the case of the Fas pathway, the FADD protein binds directly to the receptor and then, via its death effector domain (DED), recruits procaspase-8 molecules [25]. This multiprotein aggregate is termed DISC (Death-Inducing Signaling Complex) [15]. In the case of TNFR1, its binding to TNF-α initiates the formation of a complex signaling complex [13] that includes TRADD, allowing for divergent outcomes: binding of TRAF2 and RIPK1 to form complex I, thereby activating NF-κB and MAPK pathways and promoting survival and inflammation, or binding of FADD to form complex IIa, which then recruits caspase-8 and triggers apoptosis [15,25].

Activation of initiator caspase-8 occurs through the binding of FADD via its death effector domain (DED) to the analogous domain of procaspase-8 [25]. The increased local concentration of procaspase-8 within DISC promotes its homodimerization and subsequent autocatalytic cleavage to form active caspase-8 [40]. The protein c-FLIP can inhibit this process by competing with procaspase-8 for binding to FADD [25].

The effector phase depends on the cell type in which cell death occurs:

- In type I cells, such as mature lymphocytes, active caspase-8 directly cleaves effector caspases-3 and -7, executing apoptosis [40].
- In type II cells, such as hepatocytes and tumor cells, direct activation of effector caspases is insufficient [27]. Caspase-8 cleaves the BID protein to generate its truncated form tBID [12], which then translocates to mitochondria and activates BAX/BAK, causing MOMP and thus engaging the intrinsic apoptosis pathway [42].

### 2.2. Ferroptosis: Molecular Mechanisms and Regulation

#### 2.2.1. Definition and Main Characteristics

Ferroptosis is an iron-dependent form of regulated cell death that is morphologically, biochemically, and genetically distinct from apoptosis. Unlike apoptosis, ferroptosis is not accompanied by typical features such as cell shrinkage, nuclear condensation, or caspase activation; instead, it is characterized by the accumulation of lipid peroxides driven by iron-dependent oxidative stress and impairment of antioxidant defense, such as GPX4 [1].

Morphologically, ferroptosis displays a distinct mitochondrial phenotype, including reduced mitochondrial volume, increased membrane density, reduction or disappearance of cristae, and rupture of the outer mitochondrial membrane, whereas the nucleus remains intact without condensation or fragmentation [20].

During ferroptosis, mitochondria exhibit characteristic shrinkage and increased membrane density; this process is initiated by lipid peroxidation, which disrupts membrane assembly and physicochemical properties [43]. Accumulation of hydroperoxides in phospholipids disrupts ion gradients and increases membrane permeability, and the formation of “hydrophilic pores” allows ions to move freely along their concentration gradients [20]. Presumably, leakage of K^+^ (and other osmotically active substances) from the organelle causes water efflux via osmosis, resulting in the physical shrinkage of mitochondria as part of an osmotic death mechanism [43]. Concurrently, reduction or complete loss of mitochondrial cristae is a fundamental morphological hallmark of ferroptosis [27]. Under normal conditions, the interaction of cardiolipin with OPA1 is required to maintain inner membrane structure and mitochondrial fusion; however, oxidation of cardiolipin and damage to the lipid bilayer by lipid peroxidation products destabilize cristae junctions, which are regulated by OPA1 and MICOS complexes [44]. This leads to disruption and “flattening” of cristae, occurring independently of classical apoptotic signals, although it correlates with loss of bioenergetic function. The final stage of organelle damage is characterized by rupture of the outer mitochondrial membrane (OMM) [27]. Accumulation of peroxidized PUFA-containing phospholipids leads to membrane thinning and increased curvature, promoting oxidative micellization and pore formation. This process generates considerable mechanical stress, and combined with osmotic imbalance (“osmotic catastrophe”), the membrane, simultaneously stretched and weakened by lipid peroxidation, ultimately loses integrity and ruptures [45]. It should be noted that data are limited: the molecular mechanisms linking hydroperoxide accumulation to specific structural changes in mitochondria during ferroptosis require further investigation using cryo-electron tomography and spatial proteomics.

These mitochondrial changes distinguish ferroptosis from apoptosis, which typically involves nuclear shrinkage and chromatin condensation [27]. Loss of mitochondrial integrity during ferroptosis is linked to iron-dependent lipid peroxidation and ROS accumulation, which cause membrane damage [1]. Despite outer membrane rupture, the nuclear envelope retains its integrity, setting ferroptosis apart from other forms of cell death associated with nuclear destruction [46]. This unique morphology reflects the dependence of ferroptosis on iron metabolism and oxidative stress rather than on caspase activation or DNA fragmentation observed in apoptosis [47]. These features make ferroptosis a distinct regulated cell death pathway with important implications for diseases associated with oxidative damage and dysregulation of iron metabolism.

#### 2.2.2. Key Molecular Mechanisms

The central biochemical event in ferroptosis is the Fenton reaction: Fe^2+^ ions in the presence of hydrogen peroxide (H_2_O_2_) generate highly reactive hydroxyl radicals (OH•) initiating a chain reaction of peroxidation of PUFA-containing phospholipids (polyunsaturated fatty acids, PUFA), primarily arachidonic and adrenic acids esterified into phosphatidylethanolamine [48]. The incorporation of PUFAs into membrane phospholipids is mediated by the enzymes ACSL4 and LPCAT3; knockout of ACSL4 renders cells resistant to ferroptosis [49].

The main anti-ferroptotic defense system is the xCT–GSH–GPX4 axis. The xCT transporter, a heterodimer of SLC7A11/SLC3A2, imports cystine in exchange for glutamate; cystine is reduced to cysteine and incorporated into glutathione (GSH) synthesis. GPX4 is the only glutathione peroxidase capable of reducing PUFA-PL-OOH directly within membranes, using GSH as an electron donor [25,48,50]. Decreased activity of any component of this axis—for example, blockade of xCT by erastin, inactivation of GPX4 by RSL3, or depletion of GSH—creates conditions conducive to ferroptosis; however, the final outcome is determined by the activity of alternative anti-ferroptotic systems—FSP1/CoQ10, GCH1/BH4, and DHODH—as well as by the cellular context and the availability of PUFA substrates. It is precisely the multilayered nature of anti-ferroptotic defense that accounts for the pronounced variability in sensitivity of different cell types to ferroptosis [51].

In parallel, alternative anti-ferroptotic systems have been identified. FSP1 (*AIFM2*) reduces ubiquinone to ubiquinol (CoQ10H2) at the plasma membrane, providing a lipid radical trap independent of GPX4 [52]. The GCH1/BH4 system provides tetrahydrobiopterin, an endogenous inhibitor of PUFA peroxidation [53]. The multi-layered nature of anti-ferroptotic defense explains the pronounced variability in the sensitivity of different cell types to ferroptosis [50].

#### 2.2.3. Molecular Markers of Ferroptosis

Laboratory verification of ferroptosis relies on a panel of specific markers. Accumulation of PUFA hydroperoxides is detected by the fluorescent probe BODIPY-C11 using flow cytometry and mass spectrometry, with identification of specific oxidized phospholipid species [54]. Secondary products of PUFA oxidation—malondialdehyde (MDA) and 4-hydroxynonenal (4-HNE)—are widely used as indirect markers. Induction of PTGS2 (cyclooxygenase-2) is a transcriptional correlate of ferroptosis; decreased GSH and GPX4 activity confirm the involvement of this mechanism [55]. The decisive criterion remains the reversibility of death by ferrostatin-1 or liproxstatin-1—specific inhibitors of PUFA peroxidation [56].

### 2.3. Other Forms of Regulated Cell Death in Mitochondrial Dysfunction

#### Pyroptosis

Pyroptosis is a form of regulated inflammatory cell death characterized by plasma membrane rupture and the release of pro-inflammatory contents [57]. Although mitochondria have traditionally played a central role in the regulation of cell death in apoptosis, current evidence indicates that they serve as key hubs controlling the initiation and amplification of pyroptosis [58].

The relationship between pyroptosis and mitochondrial dysfunction is bidirectional: mitochondrial stress can trigger pyroptosis, and pyroptosis effectors (gasdermins) directly damage mitochondria.

Activated gasdermin fragments (GSDM-NT), which normally form pores in the plasma membrane, are also capable of targeting mitochondria, when the N-terminal fragment of gasdermin D rapidly damages both the outer and inner mitochondrial membranes. This occurs prior to substantial damage to the plasma membrane [59]. A key factor directing gasdermins to mitochondria is their high affinity for cardiolipin, a lipid specific to mitochondrial membranes [60]. The presence of cardiolipin on the outer membrane triggers an initial round of pore assembly, leading to the release of reactive oxygen species (ROS) and further damage. Gasdermin insertion into mitochondria results in loss of membrane potential, cessation of ATP synthesis, organelle fragmentation, and release of mitochondrial DNA (mtDNA) into the cytosol [61].

Disruptions in mitochondrial function generate signals that activate inflammasomes and initiate the pyroptotic cascade. Excessive Mitochondrial ROS (mtROS) production promotes activation of the NLRP3 inflammasome [58]. For example, in the *LRRK2* G2019S mutation, elevated mtROS levels direct GSDMD to mitochondria, switching the cell death modality from classical pyroptosis to necroptosis [62]. Free mtDNA in the cytosol is recognized by the sensors cGAS, AIM2, or ZBP1 [63]. This activates inflammatory pathways (e.g., cGAS-STING), enhances inflammasome activity, and facilitates the assembly of the PANoptosome—a complex integrating features of apoptosis, necroptosis, and pyroptosis [58]. In addition, agents such as metformin can induce mitochondrial dysfunction that activates the AMPK/SIRT1/NF-κB pathway, leading to caspase-3/GSDME-mediated pyroptosis of cancer cells [64].

**Figure 1:**
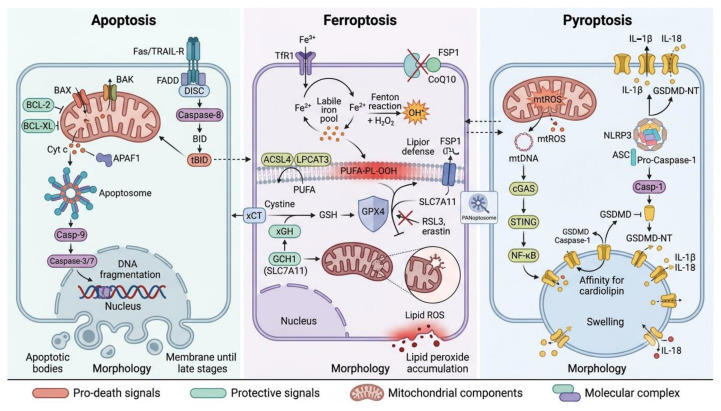
Comparative Scheme: Apoptosis vs. Ferroptosis vs. Pyroptosis—Morphology, Key Molecules, Reversibility.

A comparative schematic of these cell death types is presented in Figure 1.

## 3. Common Triggers of Apoptosis and Ferroptosis in Mitochondrial Dysfunction

### 3.1. Production of Reactive Oxygen Species by Mitochondria

The mitochondrial respiratory chain is the principal intracellular source of ROS, which are generated by electron “leakage” to oxygen to form superoxide anion (O2•^−^), subsequently converted to H_2_O_2_ [36].

The main sites of ROS generation are complexes I and III [36]. It has been shown that ROS generated at complex I in the mitochondrial matrix catalyze iron-dependent peroxidation of mitochondrial lipids, directly initiating ferroptosis, whereas ROS from complex III, released into the intermembrane space, may be less effective in this process [65]. ROS serve as a universal regulator: moderate levels of H_2_O_2_ activate redox-sensitive factors such as p53 and JNK, inducing expression of pro-apoptotic BCL-2 family proteins such as PUMA and BIM, leading to apoptosis [66]. Massive ROS release and accumulation of lipid peroxidation products cause irreversible membrane damage and ferroptotic death.

### 3.2. Disruption of Reducing Equivalent Metabolism

Mitochondrial dysfunction substantially reshapes the pools of antioxidant cofactors. Complex I deficiency reduces NADH production in the Krebs cycle, limiting NADPH regeneration via the pentose phosphate pathway. NADPH is an obligatory cofactor for glutathione reductase, which maintains GSH in its reduced state [36]. Concurrently, NADPH deficiency weakens the thioredoxin system and peroxiredoxins, depriving the cell of anti-apoptotic defense [67]. In patients with mitochondrial defects, for example, *GFM1* mutations, increased sensitivity to ferroptosis inducers is observed precisely because of this disruption of redox balance [68].

### 3.3. Imbalance of Intracellular Iron and Heme Metabolism

Mitochondria are the central hub of iron metabolism, where heme synthesis and assembly of iron-sulfur (Fe-S) clusters—cofactors of subunits of complexes I, II, and III—take place [69]. During mitochondrial dysfunction, disruption of these processes leads to accumulation of labile Fe^2+^ in the cytosol and mitochondrial matrix [70]. The labile iron pool is a direct substrate for the Fenton reaction and a direct pro-ferroptotic factor [66].

A special role is played by the interaction of released cytochrome c with H_2_O_2_. In the presence of anionic phospholipids, particularly cardiolipin, cytochrome c acquires peroxidase activity and can release free iron from its own heme (it should be noted that the primary source of labile iron in mitochondrial dysfunction is considered to be the degradation of iron–sulfur clusters of respiratory chain subunits [71,72]. Iron release from the heme of cytochrome c has been demonstrated in vitro under conditions of excess H_2_O_2_ and ionizing radiation [73]; however, the significance of this mechanism in vivo in mitochondrial diseases requires further confirmation). This creates a “vicious cycle,” in which cytochrome c release as an apoptotic signal directly activates the ferroptotic mechanism [74].

### 3.4. Disruption of Calcium Homeostasis

The mitochondrial membrane potential ΔΨm ensures uptake and buffering of cytosolic Ca^2+^. A decrease in ΔΨm during mitochondrial dysfunction disrupts this function, leading to chronic cytosolic calcium overload [75]. With moderate Ca^2+^ elevation, caspase-8 is activated via the calcium-dependent protease calpain, which cleaves BID to tBID, triggering mitochondrial apoptosis. With significant calcium overload against a background of ATP depletion, opening of mPTP occurs [76]. Thus, the intensity of calcium overload determines which cell death pathway is predominantly executed.

### 3.5. Oxidative Phosphorylation and Bioenergetic Control of Cell Death

Mitochondrial bioenergetics sets the “switches” between cell death modalities. Mitochondria establish a proton gradient and ΔΨm that is utilized by ATP synthase for ATP synthesis via oxidative phosphorylation [77]. Opening of the permeability transition pore and loss of ΔΨm are accompanied by cessation of oxidative phosphorylation, ATP collapse, and subsequent cell death [78]. Classical studies demonstrate that apoptosis requires ATP, whereas profound ATP depletion diverts the same signals toward necrosis [79]. In the pancreas under oxidative stress, low doses of H_2_O_2_, with ATP and reserve respiratory capacity still preserved, predominantly induce apoptosis, whereas high doses cause loss of ΔΨm, a decline in reserve respiratory capacity, reduced ATP, and a shift to necrosis [80]. The ATP/ADP ratio and ΔΨm serve as markers and switches: in apoptosis, a moderate drop in ATP and an increase in the ADP:ATP ratio correlate with the degree of apoptosis, whereas in necrosis the ADP:ATP ratio rises sharply (over 15), reflecting energetic collapse [81]. A review emphasizes that apoptosis often initiates with preserved or even elevated ΔΨm and ATP levels, and subsequently concludes with ΔΨm collapse and ATP decline. Regarding ferroptosis, its dependence on lipid peroxidation is well established; mitochondrial respiration and the tricarboxylic acid cycle can both enhance ROS production (e.g., via hyperpolarization and superoxide excess) and modulate sensitivity to ferroptosis [77]. Conversely, energy stress and AMPK activation can suppress ferroptosis, indicating that low ATP does not invariably lead to ferroptosis and may sometimes render it less likely [82].

Thus, the evidence strongly supports that ATP levels and ΔΨm integrity influence the choice between apoptosis and necrosis, with the ADP/ATP ratio serving as both an indicator and, in part, a switch between death modalities. For ferroptosis, the connection to bioenergetics is mediated through mitochondrial respiration, ROS, and signaling pathways (e.g., AMPK) rather than through global ATP depletion alone.

### 3.6. Mitochondrial Dynamics (Fusion/Fission) as a Regulator of Cell Fate

Mitochondrial fusion and fission shape cell fate decisions by linking morphology, bioenergetics, and activation of death pathways; the balance between DRP1-mediated fragmentation and MFN1/2–OPA1-dependent fusion directly influences apoptosis and ferroptosis.

DRP1, the key GTPase of mitochondrial fission, is recruited to the outer membrane during apoptosis, where it assembles into ring-like structures and promotes mitochondrial fragmentation and cytochrome c release [83]. Moreover, DRP1 co-localizes with BAX and Mfn2 at division sites, activates and oligomerizes BAX, thereby enhancing outer membrane permeabilization and the release of pro-apoptotic factors [84]. However, fragmentation per se is not a sufficient trigger for apoptosis: changes in morphology and regulation of death can be partially uncoupled [85]. In contrast, MFN1/2 (on the outer membrane) and OPA1 (on the inner membrane) mediate fusion, maintaining the integrity of the mitochondrial network, organelle quality, and functionality [86]. OPA1 stabilizes cristae structure and increases respiratory efficiency, preventing dysfunction and cytochrome c release under stress conditions [87]. Upon loss of membrane potential (ΔΨm), the OMA1 protease is activated and cleaves OPA1, thereby disabling fusion and leaving DRP1-mediated fission unopposed, which leads to total fragmentation and primes the cell for apoptosis or stress responses [88]. In ferroptosis, DRP1 activation, its translocation to mitochondria, network fragmentation, and loss of ΔΨm are also observed upon pharmacological induction [89]. Depletion of DRP1 or overexpression of MFN2 slows the kinetics of ferroptosis and lipid peroxidation, indicating a functional contribution of fragmentation to the acceleration of death, although these manipulations do not completely abolish ferroptosis [89]. In invertebrate models of hypoxia, Drp1/MTP18-dependent fission is also required for hypoxia-induced ferroptosis, but these data were obtained in a specific system [90].

The evidence confirms that DRP1-dependent fragmentation promotes BAX-mediated outer membrane permeabilization and apoptosis, but is not itself the sole cause of death; MFN1/2 and OPA1 maintain an integrated, energetically competent network and protect against apoptosis; in ferroptosis, DRP1 activation and fragmentation accelerate death and the accumulation of lipid hydroperoxides, yet they act rather as modulators than as an obligatory binary “switch” of ferroptosis.

### 3.7. Mitophagy: Protective Function and Pathological Consequences

PINK1/Parkin-mediated mitophagy removes depolarized mitochondria and must be tightly balanced, since both its deficiency and excess lead to cell death through distinct mechanisms. Selective mitophagy via PINK1/Parkin is activated on mitochondria with loss of membrane potential (ΔΨm), eliminating them and thereby limiting chronic stress, reactive oxygen species (ROS) production, and cell death, particularly in neurons. The mechanism involves stabilization of PINK1 on the outer membrane upon ΔΨm loss, which recruits Parkin to low-ΔΨm mitochondria and activates its E3 ligase activity, triggering autophagic degradation of these organelles [91]. This selective clearance of depolarized mitochondria prevents the accumulation of organelles that generate excess ROS and can initiate apoptosis [92]. PINK1/Parkin deficiency impairs mitophagy, leading to age-dependent accumulation of dysfunctional mitochondria, elevated ROS, and neurodegeneration, as observed in Parkinson’s disease [93]. The PINK1/Parkin pathway “finely tunes” the mitochondrial pool and bioenergetics, and age-related impairment of mitophagy enables the build-up of harmful mitochondria in various neurodegenerative disorders [94]. In Leber’s hereditary optic neuropathy (LHON), caused by a mitochondrial DNA mutation, persistently elevated autophagy and mitophagy, excess ROS, defective bioenergetics, and increased apoptosis are observed, illustrating how disrupted mitochondrial quality control results in retinal ganglion cell death [88] (direct pro-ferroptotic signaling in PINK1/Parkin deficiency has not been demonstrated in these studies; the link to ferroptosis is discussed in reviews, but without specific data for Parkinson’s disease or LHON [95]). On the other hand, excessive mitophagy and depletion of the mitochondrial pool are also detrimental. As emphasized in a review on PINK1/Parkin, this system coordinates both mitophagy and mitochondrial biogenesis; the uncoupling of one process without compensation by the other is harmful, and mitochondrial density and energy status are strictly dose-dependent parameters [96]. In LHON, sustained pathological mitophagy, combined with compensatory yet insufficient mitochondrial biogenesis, disrupts mitochondrial homeostasis, causing bioenergetic defects, excess ROS, and increased apoptosis [88]. In other models, excessive mitophagy can reduce total mitochondrial content and lead to “mitophagy-mediated death,” in which the remaining mitochondria become overloaded.

**Figure 2:**
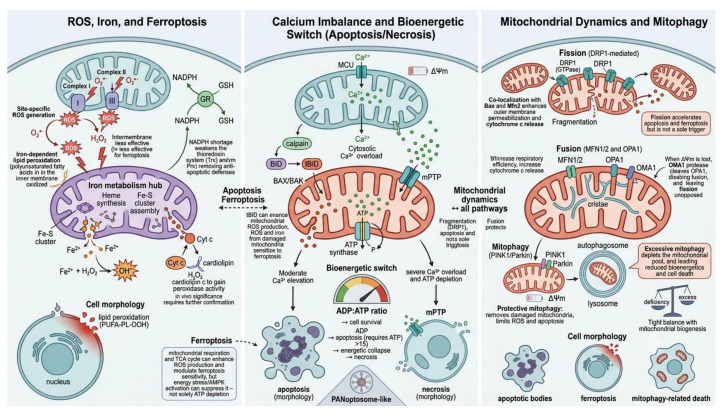
Mitochondrion as a cell death hub: complexes I–IV—ROS—branching into apoptosis/ferroptosis/pyroptosis.

The relationship of the described cell death pathways to the mitochondrion is shown in Figure 2.

## 4. Molecular Crosstalk Between Apoptosis and Ferroptosis

The triggers described in Section 3—overproduction of mtROS, calcium overload, and disrupted iron metabolism—are insufficient on their own to initiate a specific death pathway. The decisive role is played by molecular crosstalk between apoptotic and ferroptotic signals, with mitochondria serving as the central hub of this interaction.

### 4.1. Cytochrome c as an Integration Point of Apoptotic and Ferroptotic Signals

Cytochrome c occupies a unique position at the intersection of two cell death pathways. Under normal conditions, cytochrome c is an essential component of the mitochondrial electron transport chain (ETC), participating in ATP synthesis [97]. During mitochondrial stress, cytochrome c dissociates from cardiolipin in the inner membrane and is released into the cytosol through MOMP, where it initiates apoptosome formation [98].

Mitochondrial stress accompanied by an increase in reactive oxygen species and hydrogen peroxide disrupts the interaction of cytochrome c with cardiolipin (CL) and triggers its dual role in apoptosis: the protein first functions as a peroxidase and subsequently as a component of the apoptosome. Under normal conditions, cytochrome c is tightly bound to cardiolipin on the inner mitochondrial membrane, and its release into the cytosol requires a two-step process: dissociation from CL followed by outer membrane permeabilization [99]. Mitochondrially generated ROS during succinate oxidation induce simultaneous events—cytochrome c release and cardiolipin loss—with both effects being ROS-dependent and inhibited by ADP. Moreover, cytochrome c already bound to CL becomes a CL-specific peroxidase that oxidizes cardiolipin to its hydroperoxides (CL-OOH), a step required for the subsequent release of pro-apoptotic factors [100]. Because cardiolipin hydroperoxides exhibit low affinity for cytochrome c, their peroxidation leads to the dissociation of the complex and release of the protein [74]. The dual role of cytochrome c is thereby realized. In the first, peroxidase stage (within the mitochondrion), binding to CL induces a partial conformational rearrangement of the protein: the Met80 residue moves away from the heme, and cytochrome c is converted into a peroxidase-like enzyme that sharply enhances H_2_O_2_-dependent cardiolipin oxidation [101]. Notably, the CL-bound form is activated at lower H_2_O_2_ concentrations than free cytochrome c. In the second, apoptosome stage (in the cytosol), following CL-OOH formation and weakened membrane association, cytochrome c is released, binds to APAF1, assembles the apoptosome, and activates the caspase cascade [102]. Thus, mitochondrial stress and ROS initiate a sequence of events: the CL-bound peroxidase form of cytochrome c leads to cardiolipin oxidation and loss leads to dissociation and release of cytochrome c leads to participation in the apoptosome, thereby enacting its functional duality in apoptosis.

### 4.2. Role of the Phospholipid Composition of Mitochondrial Membranes in Switching Cell Death Pathways

Ferroptosis fundamentally differs from other types of cell death by excessive peroxidation of phospholipids containing PUFAs [45]. According to current models, the endoplasmic reticulum (ER) is the key initial site of lipid peroxide accumulation during ferroptosis. Lipid peroxidation begins in ER membranes and only at later stages spreads to the plasma membrane, which ultimately leads to membrane rupture and cell lysis [96].

The most significant oxidation during ferroptosis occurs in phosphatidylethanolamines (PE) containing arachidonic or adrenic acids [96]. Cardiolipin (CL) is a unique dimeric phospholipid of the inner mitochondrial membrane, accounting for 10–20% of its lipid composition and critically important for the functioning of respiratory supercomplexes [103]. In the context of apoptosis, mitochondrial phospholipase iPLA2γ oxidizes cardiolipin to hydroperoxide forms, inducing cytochrome c release and signaling mitochondrial stress. In the context of ferroptosis, oxidized cardiolipin serves as a substrate for GPX4; when GPX4 is deficient, its hydroperoxides accumulate, disrupting the membrane ion barrier [104]. Phosphatidic acid, generated by cardiolipin hydrolysis, further enhances the peroxidase activity of cytochrome c and iron release from heme.

### 4.3. Regulatory Role of the Transcription Factor p53

Transcription factor p53 occupies a central place in the integration of apoptotic and ferroptotic signals during mitochondrial stress, acting as a potent bidirectional regulator capable of both promoting and inhibiting ferroptosis depending on the context [43]. On the one hand, p53 regulates the transcription of pro-apoptotic BCL-2 family proteins, such as PUMA, NOXA, and BAX, which trigger MOMP and cytochrome c release [66]. On the other hand, the canonical mechanism of p53-mediated ferroptosis involves transcriptional repression of SLC7A11, a component of the Xc- system responsible for cystine import [105]. This leads to a decrease in GSH levels and inactivation of GPX4, causing lethal ROS accumulation [106]. In parallel, p53-dependent induction of SAT1 and ALOX12 directly activates the ferroptotic program [107]. Thus, p53 functions as a molecular “conductor” that simultaneously enhances both cell death pathways.

### 4.4. BCL-2 Family Proteins, mPTP, and Regulation of Ferroptotic Sensitivity

Although direct evidence that BCL-2 reduces electron leak specifically from complexes I and III of the respiratory chain has not been found, the body of data provides indirect support for such a mechanism—via decreased mitochondrial ROS and suppression of Fenton chemistry.

Considering the relationship between BCL-2, mitochondrial ROS, and ferroptosis, it can be noted that upon co-exposure to molybdenum and cadmium, reduced Bcl-2 in duck kidney was accompanied by increased Fe^2+^, lipid peroxidation, and decreased GPX4 and ferritin; inhibition of Bcl-2 exacerbated these changes, whereas its overexpression and a mitochondrial ROS inhibitor (ROS-IN-1) attenuated ferroptosis. This directly links the anti-ferroptotic effect of Bcl-2 to decreased mitoROS [108].

Considering the regulation of mitochondrial ROS by the respiratory chain, a mechanistic review indicates that Bcl-2 modulates complex IV activity, facilitates mitochondrial GSH transport, and interacts with Rac1 [109]. Although complexes I/III are not explicitly mentioned, a reduction in total mitoROS through more efficient electron transfer to complex IV and increased antioxidant capacity would logically diminish reverse and side electron transfer at complexes I/III, thereby lowering superoxide—the precursor of H_2_O_2_ for the Fenton reaction.

Turning to the role of BCL-2 in proton leak and redox control, it should be noted that in β-cells, blockade or knockout of Bcl-2 elevates peroxides and triggers a redox-dependent mitochondrial proton leak (partly via mPTP), which is suppressed by the antioxidant NAC. Endogenous Bcl-2 modulates ROS signaling and suppresses redox-regulated leak [110]. The reduction in excess ROS and leak indirectly points to decreased uncoupled electron transfer, where the major leak sites are complexes I and III, whose superoxide and H_2_O_2_ serve as substrates for the Fenton reaction with Fe^2+^.

Regarding ROS, lipid peroxidation, and Fenton chemistry as the core of ferroptosis, it should be emphasized that ferroptosis results from the accumulation of iron-dependent lipid hydroperoxides (LOOH) derived from ROS; mitochondrial ROS and lipid peroxidation are key drivers of the process [111].

Ferroptosis is also critically dependent on lipid metabolism enzymes such as ACSL4 and LPCAT3, which mediate the incorporation of PUFAs into membrane phospholipids [112]. Mitochondria participate in this process through β-oxidation of fatty acids, which can lower the levels of available PUFAs and thereby inhibit ferroptosis [1]. Signaling pathways such as AMPK can also limit ferroptosis by suppressing fatty acid synthesis under energy stress [113].

In summary, the assembly of the mechanism (with due consideration of data limitations) appears as follows. Based on the evidence, the following scheme is substantiated: mitochondrial BCL-2 reduces mitoROS by enhancing complex IV efficiency, boosting mitochondrial glutathione, and controlling redox-sensitive proton leak. Lower mitoROS means less generation of superoxide/hydrogen peroxide that form the Fenton substrate in the presence of Fe^2+^, thereby limiting LOOH and ferroptosis (demonstrated in the Mo/Cd model). Direct data on the influence of BCL-2 specifically on leak at complexes I/III are lacking, but the aggregate results on reduced mitoROS, respiratory chain regulation, and ferroptosis render the proposed mechanism scientifically justified.

### 4.5. The Mitochondrial Permeability Transition Pore as a Bifurcation Point of Apoptosis, Necrosis, and Ferroptosis

The mitochondrial permeability transition pore (mPTP) indeed serves as a bifurcation point among apoptosis, necrosis, and ferroptosis, with the duration and extent of opening as well as the energetic status of the cell being decisive.

The mPTP is a Ca^2+^- and ROS-sensitive channel in the inner mitochondrial membrane; its opening leads to depolarization, disruption of oxidative phosphorylation, and cell death, but transient and sustained opening have different consequences [107]. Brief, low-conductance mPTP flickers occur under moderate Ca^2+^ and ROS and can normally be reversible: transient depolarization, calcium ion discharge, and limitation of hyperpolarization and excess ROS are observed, after which mitochondria repolarize [108]. Such mPTP flickers do not necessarily cause cell death on their own until a threshold number of mitochondria with open pores is exceeded [109]. In contrast, prolonged, high-conductance opening causes loss of ΔΨm, cessation of ATP synthesis, swelling, and outer membrane rupture with the release of pro-apoptotic proteins [110]. The subsequent fate of the cell depends on ATP availability: when ATP is preserved (e.g., via glycolysis), the mPTP can participate in apoptosis through the release of cytochrome c and other factors [111], whereas when ATP drops profoundly, sustained mPTP opening leads to necrotic (unprogrammed) death [112].

In recent years, studies have directly linked the mPTP to ferroptosis. During ferroptosis, the mPTP opens, causing depolarization, mitochondrial swelling, and the release of molecules, including oxidized mitochondrial DNA; this amplifies ferroptosis through activation of the cGAS-STING pathway and ferritinophagy [114]. In model systems, tert-butyl hydroperoxide (TBH) combined with iron simultaneously triggers lipid peroxidation and mPTP opening (swelling, loss of oxidative phosphorylation), and these effects are suppressed by both lipid peroxidation inhibitors and cyclosporin A (an mPTP inhibitor), indicating the pore’s involvement in “mitochondria-dependent ferroptosis” [115].

Thus, it is emphasized in the literature that the mPTP is a central node that coordinates metabolic and redox stress and the choice of death mode (apoptosis, necrosis, ferroptosis) depending on the duration of opening, the fraction of mitochondria involved, and ATP levels.

**Figure 3:**
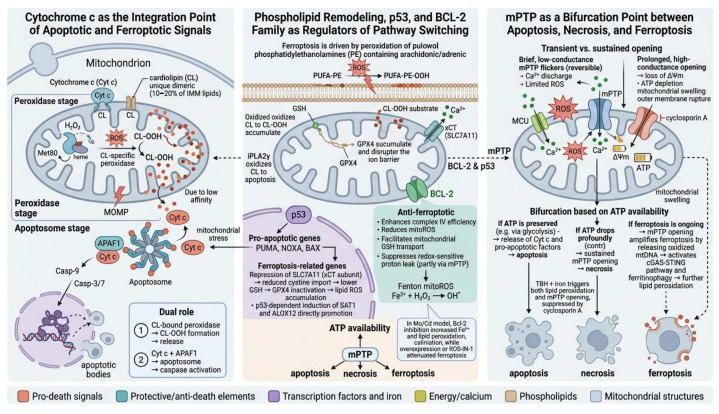
Molecular crosstalk: cytochrome c, cardiolipin, p53 as nodal points; arrows indicating pro-apoptotic/pro-ferroptotic effects.

Scheme of molecular crosstalk between apoptosis and ferroptosis is shown in Figure 3.

## 5. Tissue Specificity of Apoptosis and Ferroptosis in Mitochondrial Diseases

Tissue specificity in the context of this review denotes the selective activation of a particular regulated cell death pathway in response to mitochondrial damage of the same nature, as determined by the biochemical composition and energetic demands of a given tissue. It is governed by: (1) the PUFA content of membranes, (2) the expression levels of GPX4 and anti-apoptotic proteins of the BCL-2 family, (3) the labile iron pool, and (4) energy dependence and the ratio of mitochondria per cell.

### 5.1. Primary Mitochondrial Diseases of the CNS

The structure of the nervous tissue determines the choice of the path of cell death. Neuronal membranes contain exceptionally high concentrations of polyunsaturated fatty acids (PUFAs), such as docosahexaenoic acid (DHA) and arachidonic acid [20]. These fatty acids possess readily abstractable bis-allylic hydrogen atoms, rendering them ideal substrates for uncontrolled lipid peroxidation [28]. Neurons are characterized by relatively low levels of certain antioxidant enzymes (e.g., catalase) and a high dependence on spare respiratory capacity, which makes them highly susceptible to ROS accumulation and glutathione (GSH) depletion [20]. During ischemia–reperfusion or traumatic brain injury (TBI), an abrupt release of iron, combined with the abundance of PUFAs, initiates the ferroptotic cascade before caspase-dependent apoptotic mechanisms can be activated [20].

In Leigh syndrome, neurons of the basal ganglia and brainstem show documented signs of both apoptosis, such as cleaved caspase-3 and TUNEL-positive cells (Terminal deoxynucleotidyl transferase-mediated dUTP Nick End Labeling), and ferroptosis, including accumulation of lipid peroxidation products, decreased GPX4, and iron deposition [116]. It should be emphasized, however, that the mere presence of these markers does not constitute sufficient evidence for ferroptosis as the dominant death mechanism: this requires pharmacological rescue of cells with ferrostatin-1 or liproxstatin-1, together with the concurrent exclusion of apoptosis, necroptosis, and nonspecific oxidative necrosis. Fibroblasts from patients with Leigh syndrome and MELAS exhibit hypersensitivity to glutathione depletion and ferroptosis caused by accumulation of mitochondrial ROS (mtROS). Mutations in complex I subunits, such as *ND3* and *NDUFA1*, render cells extremely susceptible to oxidative stress [65,117].

Leber’s hereditary optic neuropathy (LHON) is the most common mitochondrial disease, associated with mutations in complex I subunits (*ND1*, *ND4*, *ND6*). The disease exclusively affects retinal ganglion cells (RGCs) and their axons. RGCs are extremely sensitive to mitochondrial dysfunction because of their unique axonal architecture and high ATP demand [118]. In this disease, pathologically enhanced mitophagy is observed in the soma of neurons, depleting the organelle pool and leading to apoptosis [119]; fibroblasts from LHON patients are hypersensitive to ferroptosis induced by buthionine sulfoximine (BSO). Mitochondria-targeted antioxidants (SkQ1) effectively block this death, confirming the role of mitochondrial lipid peroxidation [65].

### 5.2. Secondary Mitochondrial Dysfunction in Neurodegeneration (Model Data)

In Parkinson’s disease with *PINK1/Parkin* mutations, defective mitophagy control leads to the accumulation of damaged mitochondria in dopaminergic neurons of the substantia nigra [70]. Under oxidative stress conditions, dopamine can enhance ferroptosis through activation of protein kinase Cδ, which phosphorylates and inactivates components of the anti-ferroptotic system [120]. In central nervous system (CNS) injury, a sharp rise in intracellular iron occurs, leading to enhanced lipid peroxidation and neuronal death. After cerebral hemorrhage, iron release from the hematoma causes oxidative stress and edema; in models of ischemic stroke, prolonged upregulation of transferrin receptor 1 (*TFR1*) is observed, increasing iron uptake and cell death [121,122]. Reduced levels of ferritin and GSH are key factors in hippocampal neuron death via the p53/SLC7A11 axis [43].

### 5.3. Cardiomyopathies

Cardiac muscle provides an ideal environment for ferroptosis owing to the high density of mitochondria and the distinctive features of iron metabolism. Cardiomyocytes contain a vast number of mitochondria, which act as a central hub of iron metabolism and the predominant source of ROS within the cell [20].

In cardiomyopathy models, inhibition of apoptosis or necroptosis does not substantially rescue cells, whereas ferroptosis inhibitors (Fer-1) and iron chelators exert a pronounced protective effect, confirming the dominant role of ferroptosis in the heart.

DOX causes iron accumulation specifically in the mitochondria of cardiomyocytes, forming DOX-Fe^2+^ complexes that lead to specific peroxidation of mitochondrial membranes and reduced expression of the protective gene *GPX4* [123]. Mitochondria become severely deformed, swollen, and lose cristae [124].

In cardiac ischemia–reperfusion (I/R), ferroptosis is activated predominantly during the reperfusion phase, when a sharp burst of ROS occurs and non-heme iron accumulates. Opening of mPTP and loss of membrane potential accompany this process, facilitating the release of death factors [125].

Therapeutic targets in ferroptosis-associated cardiomyopathies include ferrostatin-1 and iron chelators, such as dexrazoxane, which effectively prevent cardiomyocyte death in models of I/R and DOX toxicity [113]. The mitochondria-targeted antioxidant MitoTEMPO has demonstrated significant cardiac protection, confirming the key role of mitochondrial lipid peroxidation [126].

### 5.4. Skeletal Muscle Damage

The tissue specificity of apoptosis and ferroptosis in skeletal muscle is determined by the unique structure of this tissue as a multinucleated syncytium, its high metabolic activity, and the presence of a specific pool of stem cells, satellite cells [127].

In different myopathies, apoptosis and ferroptosis have distinct features. In Duchenne muscular dystrophy, the absence of dystrophin leads to sarcolemmal instability and calcium overload, which activates the BAX/Bak-dependent mitochondrial pathway of apoptosis [127], while accumulation of free iron and peroxides triggers the Fenton reaction, generating hydroxyl radicals that disrupt the lipid bilayer of the sarcolemma [128]. In sarcopenia, an imbalance of Bcl-2 family proteins and activation of AIF and endonuclease G, which migrate from mitochondria to nuclei, cause DNA fragmentation via a caspase-independent pathway [129]; in ferroptotic processes, age-related decline of transferrin receptor 1 (Tfr1) paradoxically leads to compensatory iron accumulation through the Zip14 (Slc39a14) transporter, triggering the ferroptosis cascade in myoblasts via the p53/SLC7A11 axis [128].

Tissue specificity manifests in a close intertwining of death mechanisms, often termed “PANoptosis,” which combines apoptosis, necroptosis, and pyroptosis. Here, mitochondrial dysfunction acts as a common triggering factor, in which ROS release stimulates lipid oxidation—i.e., ferroptosis—and simultaneously opens mPTP, releasing apoptotic factors [127]. Various regulatory proteins, such as p53, can simultaneously suppress antioxidant defense (*SLC7A11*), promoting ferroptosis, and activate pro-apoptotic genes (*BAX*, *PUMA*) [130].

Mitochondrial dysfunction in skeletal muscle is often coupled with disrupted iron metabolism. In cellular models of nemaline myopathy caused by *ACTA1* and *NEB* mutations, iron and lipofuscin accumulation as well as enhanced lipid peroxidation in mitochondria have been detected, as deficiency of frataxin (FXN) and ISCU proteins disrupts iron-sulfur cluster assembly, leading to excess free iron in mitochondria and triggering the ferroptotic cascade [131].

### 5.5. Hepatocellular Manifestations and mtDNA Depletion Syndromes (MDS)

The liver is the main iron storage organ, making it a critical organ for studying ferroptotic mechanisms. Hepatocytes play a central role in GSH biosynthesis, iron metabolism, and ROS detoxification; therefore, in mitochondrial dysfunction, the liver is affected through several parallel mechanisms. ACSL4 protein is a key factor for sensitivity of hepatocellular carcinoma (HCC) cells to ferroptosis, enriching membranes with PUFAs. At the same time, ACSL4 deficiency can reduce the level of liver fibrosis despite suppressing ferroptosis [112].

mtDNA depletion syndromes (MDS) represent a group of severe genetic disorders characterized by a drastic reduction in mtDNA copy number in affected organs and tissues [1]. The main mechanism of damage in MDS is impaired respiratory chain function, leading to ATP deficiency and excessive ROS production, which act as triggers for apoptosis [27]. When excess iron and glutathione deficiency are present in hepatocytes, both apoptosis and ferroptosis are activated [131]. Mutations in the deoxyguanosine kinase gene *DGUOK*, causing mitochondrial DNA depletion syndrome, render the liver particularly susceptible to ferroptosis due to respiratory chain defects and free radical accumulation. Under conditions of mtDNA depletion and accompanying respiratory dysfunction, the liver becomes critically sensitive to iron-dependent ferroptosis, especially when free iron accumulates [1], which creates conditions predisposing to ferroptosis; however, direct evidence of ferroptosis as the cause of hepatocyte death in DGUOK-MDS—obtained with specific inhibitors and morphological verification—is currently limited. The Nrf2 pathway plays an important role in hepatic protection, controlling the expression of antioxidant enzymes and iron metabolism proteins [112].

Deficiency of mitochondrial thymidine kinase 2 (Tk2) leads to mtDNA depletion, which *in vivo* models manifest as adipose tissue hypotrophy and impaired fat accumulation, underscoring the effect of MDS on lipid metabolism in specific depots [44]. Stress caused by mtDNA damage in MDS can activate the cGAS-STING pathway, triggering autophagy-dependent ferroptosis [107].

Summary is shown in Table 1.

**Figure 4:**
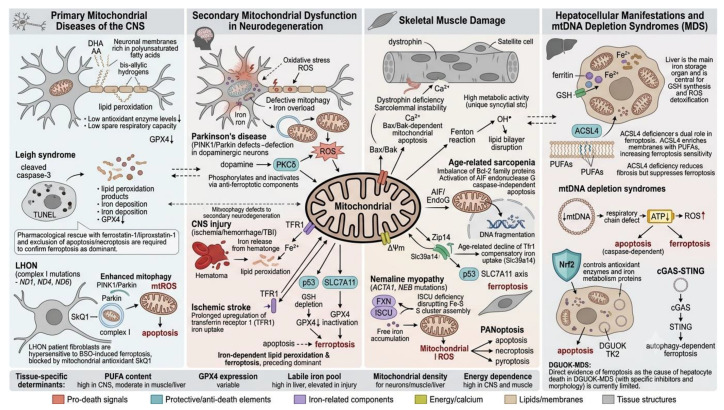
Tissue-specific map: four tissue types with indication of the dominant death pathway and its molecular basis.

And the tissue specificity of apoptosis and ferroptosis in mitochondrial diseases is shown schematically in Figure 4.

## 6. Diagnostic Criteria and Methods for Identifying Cell Death Types

### 6.1. Morphological Criteria

The main changes during ferroptosis are localized in mitochondria, which become reduced in size and shrunken, have increased membrane density, a reduced number or complete absence of mitochondrial cristae, and rupture of the outer membrane [20]. The nuclear structure remains virtually unchanged: there is no chromatin condensation or nuclear fragmentation. At the final stage, ferroptosis exhibits features of necrotic morphology, including plasma membrane rupture and cell swelling (oncosis), leading to release of contents into the extracellular space [113]. In contrast, apoptosis is characterized by cell rounding, pronounced chromatin condensation (pyknosis), nuclear fragmentation, cytoplasmic shrinkage, and formation of apoptotic bodies [27]. Organelles generally remain intact until late stages, and the plasma membrane forms characteristic protrusions—blebbing.

### 6.2. Biochemical and Molecular Markers

For ferroptosis, the key feature is the accumulation of lipid hydroperoxides (LOOH), especially in phosphatidylethanolamine (PE) species containing PUFAs [1]. The end products of this process are toxic aldehydes—MDA and 4-HNE [113]. In addition, an increase in the pool of free redox-active iron (Fe^2+^) in the cytosol (LIP) and transferrin receptor (TFR1), decreased levels of ferritin (*FTH1/FTL*), decreased level or activity of GPX4, decreased expression of *SLC7A11*—which leads to cysteine deficiency and glutathione depletion—increased expression of acyl-CoA synthetase 4 (ACSL4), which incorporates PUFAs into membranes making them vulnerable to oxidation, and increased expression of *PTGS2* are characteristic [1,12,105,113].

The main biochemical marker of apoptosis is the presence of active initiator (caspase-8, -9) and executioner (caspase-3, -6, -7) proteases, with subsequent release into the cytoplasm of proteins from the mitochondrial intermembrane space: cytochrome c, SMAC/DIABLO, and OMI/HTRA2, increased levels of pro-apoptotic factors (BAX, BAK, PUMA, BIM) with simultaneous decrease or neutralization of anti-apoptotic proteins (BCL-2, BCL-XL, MCL-1). Terminal markers include DNA degradation and substrates, such as DNA fragmentation detected by the TUNEL assay, cleavage of caspase protein substrates like PARP1, and externalization of membrane phosphatidylserine, enabling phagocytic clearance of the cell without inflammation [12,15,25,26].

The markers are shown in Table 2.

The listed markers (active caspases, the BAX/BCL-2 ratio, MDA) are general markers of apoptosis or ferroptosis and are not specific to mitochondrial diseases as a nosological group. Their diagnostic value is realized only in combination with genetic confirmation of an mtDNA/nDNA mutation and functional assays of OXPHOS activity.

### 6.3. Pharmacological Differentiation

A fundamentally important tool for verifying the type of cell death is the use of highly selective inhibitors. Cell death should be completely or significantly prevented by lipophilic radical-trapping antioxidants, such as ferrostatin-1 (Fer-1) and liproxstatin-1 (Lip-1), or by iron chelators, such as deferoxamine (DFO) or dexrazoxane [124]. It is important that ferrostatin-1 blocks lipid ROS in ferroptosis but does not affect their generation in non-canonical pyroptosis [45]. Ferroptosis is a caspase-independent process and is therefore insensitive to pan-caspase inhibitors (e.g., zVAD-fmk), which block apoptosis [27]. Ferroptosis is also not suppressed by necroptosis inhibitors such as necrostatin-1 (Nec-1) [125].

### 6.4. Criteria for Verification of Ferroptosis: From Biomarkers to Demonstration of Causality

It is fundamentally important to distinguish between indirect markers of oxidative stress and evidence of ferroptosis as the cause of cell death. Lipid peroxidation, elevated MDA/4-HNE, decreased GPX4, and iron accumulation are also observed in oxidative necrosis, apoptosis with secondary oxidative damage, and pyroptosis. Minimal verification criteria for ferroptosis include: (1) pharmacological rescue by specific lipid radical trapping agents—ferrostatin-1 or liproxstatin-1; (2) exclusion of apoptotic contribution (resistance to zVAD-fmk) and necroptotic contribution (resistance to necrostatin-1); (3) identification of specific oxidized phospholipid species (PE-AA-OOH, PE-AdA-OOH) by lipidomics/mass spectrometry. In the majority of cited studies on primary mitochondrial diseases, the complete set of these criteria has not been fulfilled, which calls for caution when interpreting the role of ferroptosis in pathogenesis.

## 7. Therapeutic Perspectives

### 7.1. Rationale for a Combined Therapeutic Strategy

The heterogeneity of cell death mechanisms in mitochondrial diseases makes monotherapy directed exclusively at one death pathway inherently inadequate. Caspase inhibitors and BCL-XL stabilizers can slow chronic cell loss through apoptosis; however, when active ferroptosis is present, their use is insufficient. Similarly, anti-ferroptotic agents, such as ferrostatin-1 and vitamin E, do not prevent MOMP-induced apoptosis. In models of myocardial ischemia, combined application of the iron chelator Deferiprone (DFP) with the antioxidant *N*-Acetylcysteine (NAC) showed better results in restoring cardiac function than monotherapy [107]. The combination of iron chelators such as deferasirox with mitochondrial pore modulators like cyclosporin A is also promising [125].

### 7.2. Potential Therapeutic Agents

Mitochondria-targeted antioxidants MitoQ and SkQ1 concentrate in the mitochondrial matrix due to covalent conjugation with the triphenylphosphonium cation, providing 100- to 1000-fold accumulation compared with the cytosol. In cellular and animal models of mitochondrial dysfunction, MitoQ reduces ROS production, prevents cardiolipin peroxidation, and thereby inhibits both apoptotic death by stabilizing cytochrome c and ferroptotic death by reducing the labile iron pool and PUFA peroxidation [50].

Cyclosporin A inhibits cyclophilin D, a regulator of mPTP opening, and prevents the transition from apoptosis to necrosis during calcium overload. Iron chelators—deferoxamine and deferiprone—directly limit ferroptotic death; deferiprone additionally exhibits a neuroprotective effect in Parkinson’s disease and LHON in phase II clinical trials [132]. NAC, a cysteine precursor, restores the GSH pool and protects against ferroptosis in mitochondrial dysfunction; its long-term safety has been convincingly demonstrated in clinical practice [133]. Apomorphine suppresses ferroptosis in patient fibroblasts through receptor-independent antioxidant mechanisms and is considered a promising candidate for clinical trials in mitochondrial myopathies [117].

### 7.3. Personalized Approach to Therapy

A key condition for rational therapy is the determination of the dominant cell death pathway in a specific patient. This requires a comprehensive diagnostic algorithm: biopsy of the affected tissue with multiparametric analysis of apoptosis markers (cleaved caspase-3, TUNEL) and ferroptosis markers (4-HNE adducts, oxidized phospholipids, GPX4); measurement in blood plasma of GSH, MDA, and biomarkers of oxidative stress; functional testing on cultured primary patient fibroblasts using specific inducers and inhibitors of both death pathways [134].

Drugs must be delivered precisely to affected tissues to avoid systemic toxicity, as processes that kill tumor cells can induce ferroptosis in healthy kidneys or liver [135]. Differences in cell death regulation between healthy and affected tissues define the therapeutic window and risk of side effects [28]. Thus, personalization of treatment entails moving from general schemes to analysis of the molecular profile of a specific tissue and monitoring of dynamic markers of oxidative stress and membrane damage.

## 8. Conclusions and Future Perspectives

Mitochondrial diseases are complex pathological states with multiple interconnected mechanisms of cell death. In this context, apoptosis and ferroptosis act not as isolated processes but as complementary pathways. They are united by common triggers—critical accumulation of mitochondrial ROS, depletion of the GSH pool, and disruption of iron homeostasis—and by points of molecular crosstalk (p53, cytochrome c and cardiolipin, BCL-2 proteins).

The pronounced tissue specificity, for example, selective vulnerability of retinal ganglion cells in LHON or cardiomyocytes under iron overload, necessitates a personalized approach, in which diagnostics should rely on the integration of genetic profiling (mtDNA and exome) and functional tests such as monitoring the dynamics of LIP (labile iron pool) and mitochondrial lipid peroxidation. It must be acknowledged that the evidence base for the causal role of ferroptosis in PMDs remains largely indirect. The majority of available data have been obtained in cell models using pharmacological inducers (erastin, RSL3) that do not reproduce the genetic nature of the mitochondrial defect, or based on immunohistochemical markers of lipid peroxidation without functional verification of ferroptosis. A priority is to conduct studies directly on tissue biopsies from patients with PMDs, employing cryo-electron microscopy and spatial lipidomics.

A new therapeutic horizon is opened by a strategy of combined inhibition, including mitochondria-targeted antioxidants (SkQ1, MitoTEMPO, MitoQ) that block lipid peroxidation directly at its site of initiation; modulators of the xCT–GPX4 axis and specific agents such as EPI-743 (vatiquinone), which have demonstrated efficacy in rescuing cells of patients with Leigh syndrome and mitochondrial epilepsy; and iron chelators (deferoxamine, dexrazoxane) and mPTP inhibitors (cyclosporin A) that prevent the massive release of pro-death factors.

Priority tasks in mitochondrial medicine remain the elucidation of the mechanisms of “switching” between death pathways and the development of systems for targeted drug delivery to organelles, which will minimize systemic toxicity and improve patient survival.

## Figures and Tables

**Table 1:** Tissue-Specific Mechanisms of Ferroptosis and Apoptosis in Mitochondrial Dysfunction.

Tissue/Disorder	Mechanisms & Findings	Key Molecules/Pathways
Neurodegenerative (Leigh syndrome, Parkinson’s, CNS injury, LHON)	– High O_2_ consumption, PUFA-rich membranes, low antioxidant enzymes. – Apoptosis (caspase-3, TUNEL) and ferroptosis (lipid peroxidation, iron deposition, ↓ *GPX4*). – Defective mitophagy (*PINK1/Parkin*) → damaged mitochondria. – Dopamine enhances ferroptosis via PKCδ. – After hemorrhage/ischemia: iron release, ↑ *TFR1*, ↑ lipid peroxidation. – LHON: complex I mutations, enhanced mitophagy, apoptosis, ferroptosis sensitivity	– *GPX4*, TUNEL, cleaved caspase-3 – *PINK1*, *Parkin* – PKCδ, *TFR1*, p53/SLC7A11 – BSO, SkQ1 (mitochondrial antioxidant)
Cardiomyopathies (Doxorubicin toxicity, I/R injury)	– DOX: iron accumulation in mitochondria → DOX-Fe^2+^ complexes → mitochondrial membrane peroxidation, ↓ *GPX4*, swollen mitochondria. – I/R: ROS burst, non-heme iron accumulation, mPTP opening, loss of membrane potential	– DOX, *GPX4* – mPTP – Ferrostatin-1, dexrazoxane, MitoTEMPO
Skeletal Muscle Damage (Duchenne MD, sarcopenia, nemaline myopathy)	– Multinucleated syncytium, high metabolic activity, satellite cells. – Duchenne: dystrophin loss → Ca^2+^ overload → BAX/Bak apoptosis; also iron/peroxides → Fenton reaction. – Sarcopenia: AIF, endonuclease G (caspase-independent); age-related Zip14 iron uptake → p53/SLC7A11 ferroptosis. – “PANoptosis” (apoptosis + necroptosis + pyroptosis) triggered by mitochondrial ROS. – Nemaline myopathy: frataxin/ISCU deficiency → iron accumulation, lipid peroxidation	– Dystrophin, BAX/Bak – AIF, endonuclease G, Zip14, p53/SLC7A11 – Frataxin (FXN), ISCU
Hepatocellular Manifestations & mtDNA Depletion Syndromes (MDS)	– Liver: major iron storage, GSH synthesis, ROS detoxification. – ACSL4 enriches membranes with PUFAs → ferroptosis sensitivity; ACSL4 deficiency reduces fibrosis. – MDS (e.g., *DGUOK*, *TK2* mutations): ↓ mtDNA → respiratory chain defects → ATP deficiency, ROS → apoptosis and ferroptosis. – Nrf2 pathway protects via antioxidant/iron metabolism genes. – cGAS-STING pathway activates autophagy-dependent ferroptosis in MDS	– ACSL4, PUFAs – *DGUOK*, *TK2* – Nrf2 – cGAS-STING

→ indicate a consequence; ↑ indicates an increase in gene or protein expression/the intensity of the process/the amount of substance; ↓ indicates a decrease in gene or protein expression/the intensity of the process/the amount of substance.

**Table 2:** Markers of Cell Death, Their Specificity for Mitochondrial Damage, and Limitations.

Marker	Specificity for Mitochondrial Damage	Limitations (Non-Specificity)
Cytochrome c in the cytoplasm	Specific for mitochondrial apoptosis (MOMP)	Not observed in extrinsic apoptosis or ferroptosis
SMAC/DIABLO, OMI/HTRA2 in the cytoplasm	Specific for mitochondrial apoptosis (release from the intermembrane space upon MOMP)	Not involved in ferroptosis or necroptosis
BAX, BAK (increased activity)	Specific for mitochondrial apoptosis (direct inducers of MOMP)	May be activated to a limited extent in some forms of necroptosis
PUMA, BIM (increased levels)	Specific for mitochondrial apoptosis (upstream inducers of BAX/BAK)	Not specific to other death types; can increase under general cellular stress
BCL-2, BCL-XL, MCL-1 (decreased or inactivated)	Specific to the regulation of mitochondrial apoptosis	Changes can be secondary to many types of stress
Caspase-8, -9 (active forms)	Caspase-9—specific for mitochondrial apoptosis; caspase-8—for extrinsic pathway	Caspases-3, -6, -7 are common to both apoptosis pathways but absent in ferroptosis
PARP1 (cleaved form)	Not specific—a substrate of caspases-3/7, thus a general apoptosis marker	Not typical for ferroptosis; can be cleaved in some necrotic processes
TUNEL (DNA fragmentation)	Not specific—an endpoint marker for many death modalities	Positive in apoptosis, secondary necrosis, and some types of necroptosis
Phosphatidylserine externalisation	Not specific—a marker of apoptosis and immune clearance	Can be exposed in necroptosis and ferroptosis (though less common)
LOOH (lipid hydroperoxides)	Not specific—a marker of oxidative stress	Positive in ferroptosis, but also in autophagy and necroptosis
MDA, 4-HNE	Not specific—general markers of lipid peroxidation	Increase in any condition involving oxidative stress, not only ferroptosis
*ACSL4* (increased expression)	Not specific for mitochondria—involved in ferroptosis	Can be induced by inflammation and lipid metabolism disorders
GPX4 (decreased level or activity)	Not specific—a key marker of ferroptosis	Not directly linked to mitochondrial damage
SLC7A11 (decreased), glutathione depletion	Not specific—metabolic markers of ferroptosis	Observed in oxidative stress from various causes
TFR1 (increased), ferritin (decreased)	Not specific—reflect disturbed iron metabolism	Characteristic of ferroptosis, but can also change in anaemia and inflammation
*PTGS2* (increased expression)	Not specific—a marker of ferroptosis and inflammation	Induced in many stress and inflammatory conditions
**Mitochondrion-specific biomarkers**		
Cardiolipin and its oxidised forms (CL, CLox)	Unique marker of mitochondrial stress (cardiolipin is present only in the inner mitochondrial membrane)	Oxidised cardiolipin can be released during mitochondrial dysfunction, but is not typical for ferroptosis or apoptosis without mitochondrial involvement
mtDNA in plasma/cytoplasm	Marker of mitochondrial cell death (release upon membrane permeabilisation)	Not specific for the death pathway (apoptosis, necrosis, ferroptosis with mitochondrial fragmentation), but strictly indicates mitochondrial damage
TFAM (decreased or released)	Functional marker of mitochondrial integrity and copy number	Decrease can be seen in general mitochondrial biogenesis defects; not specific to any particular death pathway
ΔΨm—measured in primary fibroblasts	Functional marker of mitochondrial health; ΔΨm drop is an early event in mitochondrial damage	Loss of potential occurs in apoptosis, necrosis, ferroptosis, ischaemia, and toxic exposures—not specific to a single death type

## Data Availability

No new data were created or analyzed in this study. Data sharing is not applicable to this article.

## Abbreviations

The following abbreviations are used in this manuscript:

4-HNE	4-hydroxynonenal
ACTA1	actin alpha 1 (skeletal muscle)
AIF	apoptosis-inducing factor
AIFM2	apoptosis-inducing factor mitochondria-associated 2 (also FSP1)
AMPK	AMP-activated protein kinase
ANT	adenine nucleotide translocator
APAF1	apoptotic protease-activating factor 1
ATP	adenosine triphosphate
BAD	BCL-2-associated agonist of cell death
BAK	BCL-2 antagonist/killer
BAX	BCL-2-associated X protein
BCL-2	B-cell lymphoma 2
BCL-W	BCL-2-like protein 2
BCL-XL	BCL-2-related protein XL
BFL-1	BCL-2-related protein A1
BH	BCL-2 homology domain
BH3	BCL-2 homology domain 3
BID	BH3-interacting domain death agonist
BIK	BCL-2-interacting killer
BIM	BCL-2-like protein 11 (BCL2L11)
BMF	BCL-2-modifying factor
BSO	buthionine sulfoximine
Ca^2+^	calcium ion
CAD	caspase-activated DNase
c-FLIP	cellular FLICE (FADD-like IL-1β-converting enzyme)-inhibitory protein
cGAS-STING	cyclic GMP-AMP synthase—stimulator of interferon genes pathway
CL	cardiolipin
CNS	central nervous system
CoQ10H_2_	ubiquinol (reduced coenzyme Q10)
CsA	cyclosporin A
dATP	deoxyadenosine triphosphate
DD	death domain
DED	death effector domain
DFP	deferiprone
DFO	deferoxamine
DGUOK	deoxyguanosine kinase
DHA	docosahexaenoic acid
DIABLO	direct IAP-binding protein with low pI (also SMAC)
DISC	death-inducing signaling complex
DNA	deoxyribonucleic acid
DOX	doxorubicin
DR	death receptor
EndoG	endonuclease G
EPI-743	vatiquinone
ER	endoplasmic reticulum
ETC	electron transport chain
FADD	Fas-associated death domain protein
FasL	Fas ligand
Fe^2+^	ferrous iron
Fer-1	ferrostatin-1
Fe-S	iron-sulfur cluster
FSP1	ferroptosis suppressor protein 1 (AIFM2)
FTH1	ferritin heavy chain 1
FTL	ferritin light chain
FXN	frataxin
GCH1	GTP cyclohydrolase 1
GFM1	G elongation factor mitochondrial 1
GPX4	glutathione peroxidase 4
GSH	glutathione (reduced)
H_2_O_2_	hydrogen peroxide
HCC	hepatocellular carcinoma
HRK	harakiri (BCL-2-interacting protein)
HtrA2	high temperature requirement protein A2 (also Omi)
I/R	ischemia–reperfusion
ICAD	inhibitor of caspase-activated DNase
iPLA2γ	calcium-independent phospholipase A2 gamma
ISCU	iron-sulfur cluster assembly enzyme
JNK	c-Jun *N*-terminal kinase
LHON	Leber’s hereditary optic neuropathy
LIP	labile iron pool
Lip-1	liproxstatin-1
LOOH	lipid hydroperoxides
LPCAT3	lysophosphatidylcholine acyltransferase 3
MAPK	mitogen-activated protein kinase
MCL-1	myeloid cell leukemia 1
MDA	malondialdehyde
MDS	mtDNA depletion syndromes
MELAS	mitochondrial encephalomyopathy, lactic acidosis, and stroke-like episodes
MitoQ	mitoquinone mesylate (mitochondria-targeted antioxidant)
MitoTEMPO	mitochondria-targeted TEMPO (antioxidant)
MLKL	mixed lineage kinase domain-like pseudokinase
MOMP	mitochondrial outer membrane permeabilization
mPTP	mitochondrial permeability transition pore
mtDNA	mitochondrial DNA
mtROS	mitochondrial reactive oxygen species
NAC	*N*-acetylcysteine
NADH	nicotinamide adenine dinucleotide (reduced)
NADPH	nicotinamide adenine dinucleotide phosphate (reduced)
NCCD	Nomenclature Committee on Cell Death
ND1, ND3, ND4, ND6	NADH dehydrogenase subunits 1, 3, 4, 6 (mitochondrial complex I)
NDUFA1	NADH:ubiquinone oxidoreductase subunit A1
Nec-1	necrostatin-1
NEB	nebulin
NF-κB	nuclear factor kappa-light-chain-enhancer of activated B cells
NOXA	phorbol-12-myristate-13-acetate-induced protein 1 (PMAIP1)
Nrf2	nuclear factor erythroid 2-related factor 2
nDNA	nuclear DNA
O_2_•^−^	superoxide anion
OH•	hydroxyl radical
Omi	serine protease Omi (also HtrA2)
OXPHOS	oxidative phosphorylation
p53	tumor protein p53
PANoptosis	combination of pyroptosis, apoptosis, and necroptosis
Parkin	E3 ubiquitin-protein ligase parkin
PARP1	poly(ADP-ribose) polymerase 1
PE	phosphatidylethanolamine
PINK1	PTEN-induced putative kinase 1
PTGS2	prostaglandin-endoperoxide synthase 2 (cyclooxygenase-2)
PUFA	polyunsaturated fatty acid
PUMA	p53-upregulated modulator of apoptosis
RCD	regulated cell death
RGC	retinal ganglion cell
RIPK1	receptor-interacting serine/threonine-protein kinase 1
RIPK3	receptor-interacting serine/threonine-protein kinase 3
ROS	reactive oxygen species
RSL3	RAS-selective lethal 3 (ferroptosis inducer)
SAT1	spermidine/spermine N1-acetyltransferase 1
SkQ1	plastoquinonyl-decyltriphenylphosphonium (mitochondria-targeted antioxidant)
SLC3A2	solute carrier family 3 member 2
SLC7A11	solute carrier family 7 member 11 (xCT)
SLC39A14	solute carrier family 39 member 14 (Zip14)
SMAC	second mitochondria-derived activator of caspases
tBID	truncated BID
TFR1/Tfr1	transferrin receptor 1
Tk2	thymidine kinase 2 (mitochondrial)
TNFα	tumor necrosis factor alpha
TNFR	tumor necrosis factor receptor
TRADD	TNFRSF1A-associated via death domain
TRAF2	TNF receptor-associated factor 2
TRAIL	TNF-related apoptosis-inducing ligand
TUNEL	terminal deoxynucleotidyl transferase dUTP nick end labeling
XIAP	X-linked inhibitor of apoptosis protein
xCT	cystine/glutamate antiporter subunit (SLC7A11)
Zip14	Zrt- and Irt-like protein 14 (SLC39A14)
zVAD-fmk	benzyloxycarbonyl-Val-Ala-Asp(OMe)-fluoromethylketone (pan-caspase inhibitor)
ΔΨm	mitochondrial membrane potential
